# Supplementary material for: College students’ stress and health in the COVID-19 pandemic: The role of academic workload, separation from school, and fears of contagion
Source: PLoS One. 2021 Feb 10;16(2):e0246676. doi: 10.1371/journal.pone.0246676 (PMC7875391; doi:10.1371/journal.pone.0246676)
Supplement: S1 Appendix — (DOCX) [file pone.0246676.s001.docx]

**S1 Appendix**

**Academic workload**

1. I am spending a lot of time thinking about how this semester’s grades could negatively affect my educational and career goals.

2. I am worrying a great deal about the effect this semester’s grades will have on my future.

3. I find myself very concerned about the grades I am likely to receive this semester.

**Separation from school**

1. I find it difficult to allow myself to depend on my group.

2. I sometimes worry that I will be hurt if I allow myself to become too close to my group.

3. I am nervous when my group gets too close.

4. My desire to feel completely at one sometimes scares my group away.

5. I prefer not to depend on my group or to have my group depend on me.

6. I often worry that my group does not really accept me.

7. I am comfortable not being close to my group.

8. I often worry my group will not always want me as a member.

9. I am somewhat uncomfortable being close to my group.

10. My group is never there when I need it.

11. I find it difficult to completely trust my group.

12. I find my group is reluctant to get as close as I would like.

13. I am not sure that I can always depend on my group to be there when I need it.

14. I sometimes worry that my group doesn't value me as much as I value my group.

15. I want to be emotionally close with my group, but I find it difficult to trust my group completely or to depend on my group.

**Fears of contagion**

1. In public, I don't care about touching the door handle without protection.

2. In public, I don’t mind sitting in a chair that has just been sat on.

3. In an elevator, I don’t mind pushing a button without protection.

4. When I’m in a crowded place, I don't worry about coronavirus from other people.

5. I don't worry about infection if other people don't wear masks.

6. Wearing a mask would make me feel safe.

**Perceived stress**

1. In the last month, how often have you been upset because of something that happened unexpectedly?

2. In the last month, how often have you felt that you were unable to control the important things in your life?

3. In the last month, how often have you felt nervous and “stressed”?

4. In the last month, how often have you felt confident about your ability to handle your personal problems?

5. In the last month, how often have you felt that things were going your way?

6. In the last month, how often have you found that you could not cope with all the things that you had to do?

7. In the last month, how often have you been able to control irritations in your life?

8. In the last month, how often have you felt that you were on top of things?

9. In the last month, how often have you been angered because of things that were outside of your control?

10. In the last month, how often have you felt difficulties were piling up so high that you could not overcome them?

**Physical and psychological health**

Have you recently...

1. been suffering from headache or pressure in your head?

2. had palpitation and worried that you might have heart trouble?

3. had discomfort or a feeling of pressure in your chest?

4. been suffering from shaking or numbness of your limbs?

5. lost much sleep through worry?

6. been taking things hard?

7. been getting along well with your family or friends?

8. been losing confidence in yourself?

9. been feeling nervous and strung-up all the time?

10. been feeling hopeful about your future?

11. been worried about your family or close friends?

12. felt that life is entirely hopeless?
