# Supplementary material for: College students’ stress and health in the COVID-19 pandemic: The role of academic workload, separation from school, and fears of contagion
Source: PLoS One. 2021 Feb 10;16(2):e0246676. doi: 10.1371/journal.pone.0246676 (PMC7875391; doi:10.1371/journal.pone.0246676)
Supplement: S1 File — (PDF) [file pone.0246676.s003.pdf]

Yan Shan University

School of Economics and Management

Office of Research Compliance

Date: March 1, 2020

To: Chunjiang Yang
Yashuo Chen
Aobo Chen et al.

From: Chunling Li President
Institutional Review Board Office of Research Compliance

Re: Approval of IRB 20-03-01

Title: health survey among college students during COVID-19 epidemic

This letter is notification of approval of the research project referenced above IRB approval of this research begins as of March 10, 2020 and ends on March 1, 2021.

The approved number of subjects to be enrolled is 20000. The IRB considers a participant to be enrolled once s/he signs a Consent Form. If additional participants are needed, you first must obtain permission from the IRB to increase the sample size.

The IRB requires that you report as soon as possible any unexpected adverse events (including non-serious and serious events) that occur during the study. If the research is expected to continue beyond 12 months, you must request Continuing Review and re-approval of the project least 6 weeks prior to the date of expiration date noted above.

If you plan to change the research project (number of participants, title, procedure, payment, consent form, etc.), you must submit a request detailing the proposed changes and receive IRB approval before the changes are implemented except when prompt changes are necessary to eliminate apparent and immediate hazards to the participants.

This study was reviewed in accordance with research regulations of Yanshan University School of Economics and Management. It has been determined the risk involved in this research is no more than minimal.

If you have any questions related to this approval, please call 0335-8057025 or send an email jgkyk@ysu.edu.cn
